# Quantum chemical investigation of predominant conformation of the antibiotic azithromycin in water and DMSO solutions: thermodynamic and NMR analysis

**DOI:** 10.1098/rsos.230409

**Published:** 2023-10-11

**Authors:** Isabel S. Hernandes, Haroldo C. Da Silva, Hélio F. Dos Santos, Eloah P. Ávila, Mauro V. De Almeida, Wagner B. De Almeida

**Affiliations:** ^1^ Laboratório de Química Computacional e Modelagem Molecular (LQC-MM), Departamento de Química Inorgânica, Instituto de Química, Universidade Federal Fluminense (UFF), Outeiro de São João Batista s/n, Campus do Valonguinho, 24020-141, Centro, Niterói, RJ, Brazil; ^2^ Núcleo de Estudos em Química Computacional, Departamento de Química, Universidade Federal de Juiz de Fora, Juiz de Fora, MG 36036-900, Brazil; ^3^ Departamento de Química, ICE, Universidade Federal de Juiz de Fora (UFJF), Campus Universitário, Martelos, Juiz de Fora, MG 36036-330, Brazil

**Keywords:** azithromycin, molecular dynamics, conformation, nuclear magnetic resonance chemical shifts, density functional theory calculations

## Abstract

Azithromycin (AZM) is a macrolide-type antibiotic used to prevent and treat serious infections (mycobacteria or MAC) that significantly inhibit bacterial growth. Knowledge of the predominant conformation in solution is of fundamental importance for advancing our understanding of the intermolecular interactions of AZM with biological targets. We report an extensive density functional theory (DFT) study of plausible AZM structures in solution considering implicit and explicit solvent effects. The best match between the experimental and theoretical nuclear magnetic resonance (NMR) profiles was used to assign the preferred conformer in solution, which was supported by the thermodynamic analysis. Among the 15 distinct AZM structures, conformer **M14**, having a short intramolecular C6-OH … N H-bond, is predicted to be dominant in water and dimethyl sulfoxide (DMSO) solutions. The results indicated that the X-ray structure backbone is mostly conserved in solution, showing that large flexible molecules with several possible conformations may assume a preferential spatial orientation in solution, which is the molecular structure that ultimately interacts with biological targets.

## 1. Introduction

Azithromycin (AZM), an antibiotic belonging to the macrolide family (as well as their counterparts clarithromycin and erythromycin), is used to treat infections in the lung cells of intubated patients with pneumonia [1], as well as infections of the nose and throat, such as sinus infection (sinusitis), skin infections, Lyme disease and some sexually transmitted infections. In addition to their antibiotic properties, AZM and other members of its family have recently been recognized for their antiviral activity [2,3]. Gielen *et al*. treated rhinovirus-infected human lung cells with AZM by monitoring the infection with reverse transcription polymerase chain reaction (RT-PCR) and verified that AZM can reduce the replication and release of this type of virus [4]. Moreover, AZM has been the target of studies aimed at its application in the treatment of COVID-19, both alone and in conjunction with other known antivirals, with better results when the application occurs at the beginning of the infection [5,6].

**Figure 1.**
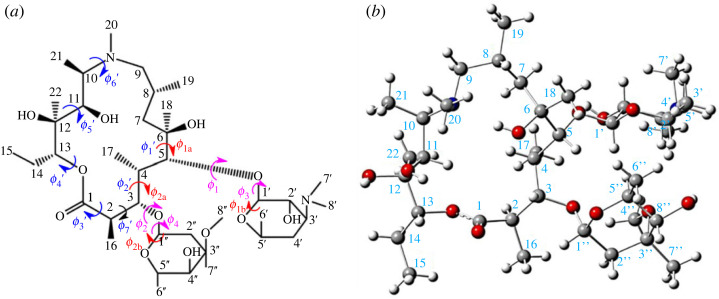
The structural formula of AZM (*a*) and the three-dimensional representation using the X-ray coordinates (*b*). The numbering scheme, and definition of important torsion angles (*φ*_i_). *φ*_1_ [C1′-O-C5-C6]; *φ*_2_ [C1″-O-C3-C4]; *φ*_3_ [O-C1′-O-C5]; *φ*_4_ [O-C1″-O-C3]; *φ*_1a_ [O-C5-C6-C7]; *φ*_2a_ [O-C3-C4-C5]; *φ*_1b_ [C2′-C1′-O-C5]; *φ*_2b_ [C2″-C1″-O-C3]; *φ*_1_′ [C4-C5-C6-C7]; *φ*_2_′ [C2-C3-C4-C5]; *φ*_3_′ [O-C1-C2-C3]; *φ*_4_′ [C12-C13-O-C1]; *φ*_5_′ [C10-C11-C12-C13]; *φ*_6_′ [C9-N-C10-C11]; *φ*_7_′ [C1-C2-C3-C4].

The structure of AZM (represented in figure 1*a*) is formed by a saturated ring of 15 atoms with two side units derived from glycides and connected to the C3 and C5 atoms. Among the saturated carbon atoms of the ring, only two, C7 and C9 (CH_2_ type), are not chiral, and the number of possible enantiomers for AZM is very high. In addition, the low unsaturation index of AZM confers, in principle, high flexibility, with glycidic ring variation drastically influencing the conformation of the molecule.

The structure of drugs in solution is of fundamental importance for their function in the biological environment. McTigue *et al*. investigated the efficiency of drugs and their association with molecular conformation. The results showed that intermolecular interactions (such as H-bonds) could be allowed or prohibited according to the geometry of the drug and protein [7]. In this context, a strong ally for conformational study in solution is nuclear magnetic resonance (NMR). Ballio *et al*., in their studies on fusicoccin conformation, used NMR to compare the conformation of several drugs of polycyclic chains in D_2_O and investigated their biological activities, which made it possible to determine the relative position of the glycidic groups of drugs [8]. More details about the molecular conformation of drugs in the solution can be obtained if the experimental NMR technique is coupled with quantum chemical calculations of magnetic shielding constants, as has been done by our group in various previous studies [9–14].

The solid-state structure of free AZM has been reported in the literature [15,16], with the absolute configurations of all asymmetric carbon atoms being precisely assigned in [16] (figure 1*b*). Previous crystallographic studies of AZM interacting with biological molecules [17–21] have also revealed the drug conformation.

The present work aims to use density functional theory (DFT) [22] methods to predict the preferred conformation of AZM in water and dimethyl sulfoxide (DMSO) solutions, using the best match between theoretical and experimental NMR profiles as a criterion.

## 2. Calculations

**Figure 2.**
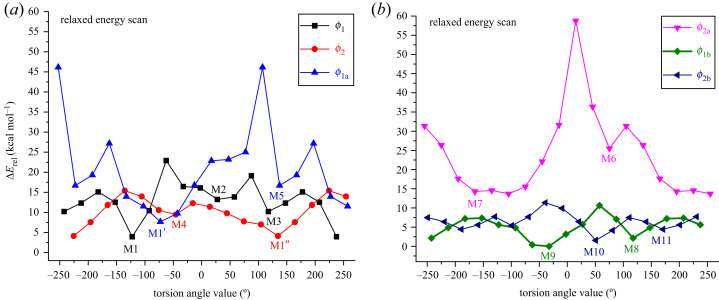
*ω*B97X-D/6–31G(d,p) relaxed potential energy curve for AZM (in the gas phase) varying torsion angles (*φ*_i_) specified below from 0° to 360° in step size of 30° (to ease visualization, torsion angles scales are in the range of −250° to + 250°). The minimum energy structures (M_i_) are indicated. Relative energies evaluated concerning the lowest energy structure M9 are shown. *φ*_1_ [C1′-O-C5-C6]; *φ*_2_ [C1″-O-C3-C4]; *φ*_1a_ [O-C5-C6-C7]; *φ*_2a_ [O-C3-C4-C5]; *φ*_1b_ [C2′-C1′-O-C5]; *φ*_2b_ [C2″-C1″-O-C3]. (*a*) *φ*_1_, *φ*_2_, *φ*_1a_ and (*b*) *φ*_1b_, *φ*_2a_, *φ*_2b_.

Using a model input, the initial structure of the AZM was constructed with pseudo-randomly chosen torsion angle values. The geometry was optimized at the DFT [22] level using the *ω*B97X-D functional [23], which carries a dispersion correction and has been found to be satisfactory for the description of thermochemistry and non-covalent interactions, using the 6–31G(d,p) basis set [24] (*ω*B97X-D/6–31G(d,p)). This optimized structure (M1) was then used to calculate the relaxed scan potential energy curves by varying the torsion angles *φ*_1_, *φ*_2_, *φ*_1a_, *φ*_2a_, *φ*_1b_, and *φ*_2b_ from 0° to 360°, with a step size of 30°, with the remaining geometrical parameters being optimized (flexible scan). The 11 stationary points, M1–M11 (In fact, M1, M1′ and M1″ structures are equivalent and have similar values for all torsion angles) found on the potential energy curves (figure 2), were further optimized at the same level of theory, and harmonic frequency calculations were performed to confirm that a true minimum energy structure was found (all frequencies were real).

In a second step, aiming to expand the conformation analysis, molecular dynamics (MD) simulations were carried out in DMSO and water solutions. For DMSO, the solvent box was built with 500 solvent molecules in a cube with an edge length of 40 Å on the side. The DMSO structure was optimized at the B3LYP/6–31G(d,p) level, and the atomic charges were calculated at the HF/6–31G(d,p) level using the ChelpG scheme. For the inter- and intra-molecular parameters, the Gaff2 force field [25,26] was applied. For H_2_O molecules, the TIP3P model [27] was used within a cubic box with a minimum solute distance and a box edge of 12 Å. The *ω*B97X-D/6–31G(d,p) optimized geometry of AZM (M1), as well as the X-ray structure [15], were used as solutes, with the Gaff2 force field used for all atoms, bonds, and angles. The simulation protocol involved optimization of the simulation box followed by heating from 50 to 298.15 K for 1500 ps in the NVT ensemble. The density was equilibrated at 298.15 K in the NPT ensemble for 600 ps, giving an average density of 1.101 ± 0.002 (DMSO) and 0.990 ± 0.004 g cm^−3^ (water), which are in good agreement with the experimental values of 1.0955 and 0.997048 g cm^−3^, respectively. Finally, 100 ns of production were run (with a time step of 2 fs), with the last 50 ns used for trajectory analysis. MD simulation was performed using the Amber 16 program [28]. The last conformations from each simulation were also included in the set of conformers optimized at the DFT level, as discussed later.

NMR calculations of shielding constants (*σ*) with chemical shifts (*δ*) determined on a δ-scale relative to tetramethylsilane (TMS) internal reference was performed using the gauge-independent atomic orbital (GIAO) method [29] with the B3LYP functional [30,31] and 6–31G(d,p) basis set (B3LYP/6–31G(d,p)), which has been shown to be adequate for organic molecules (e.g. see [32]). The solvent effect was accounted for using the polarizable continuum model (PCM) [33] for the following solvents (dielectric constants are given in parentheses): water (*ε* = 78.35) and DMSO (*ε* = 46.68). All the quantum chemical calculations were performed using the Gaussian 09 software package [34].

## 3. Results and discussion

**Figure 3.**
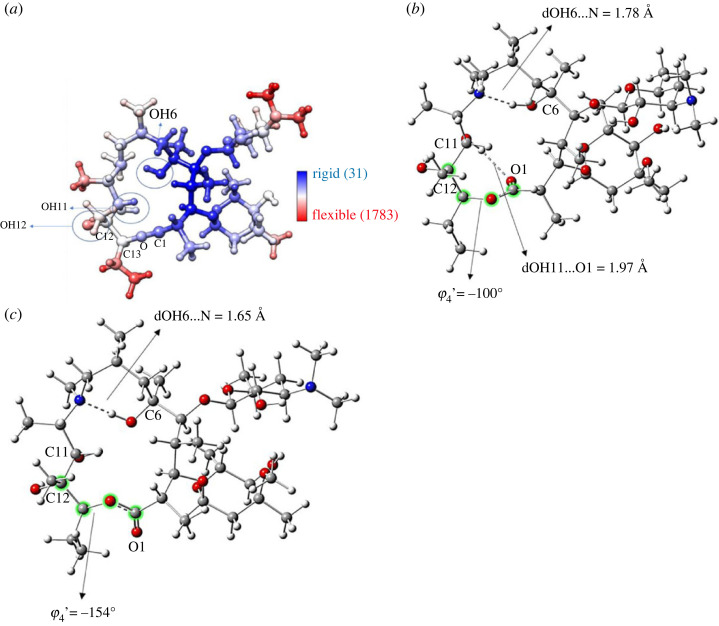
(*a*) Average atomic fluctuation calculated along 50 ns of MD trajectory in aqueous solution. The values in parenthesis are the calculated b-factor range used to colour the figure according to the flexibility of the molecular regions. The DFT-optimized structures of AZM in two distinct conformations are represented in (*b*) and (*c*), named M14 and M15 respectively. The values of torsion angles and distances were calculated in an aqueous solution within the PCM approach.

Two input structures were used in the MD simulations: the initial DFT-optimized M1 structure (water only) and the solid state from [15] (water and DMSO solutions). The conformation of AZM was monitored along the MD trajectory using the torsion angles defined in figure 1. The structural data are presented in table 1. For the simulations using the X-ray structure as the input, we observed that the average conformation of AZM in solution was in satisfactory agreement with the X-ray data, with a mean absolute deviation (MAD) less than 5° in both solvents. If the root mean square deviation (RMSD) of the whole structure was used, the values were 0.64 Å (water) and 0.62 Å (DMSO), suggesting a fairly rigid structure, even in solution under thermodynamic conditions (figure 3*a*). The final structure from the MD trajectories was optimized using the *ω*B97X-D-PCM/6–31G(d,p) protocol, and the optimized torsion angles are shown in table 1 for M12, M14 and M15 structures. The torsion angles for the X-ray structure [15] are listed in the fourth column of table 1. Electronic supplementary material, figure S1 shows a comparison between the MD average torsion angles and those obtained from the last frame of the MD simulation in water (structure M14) and DMSO (structure M15). A good match was observed, indicating that the use of the last frame from the MD simulation in DFT geometry optimization is an adequate procedure.

**Table 1.** Structural parameters (φ_i_ (°)) calculated and observed for AZM. The numbering scheme is shown in figure 1.

torsion angles (°)	M1^a^	M12^b^	X-rays	MD average structure in water	M14^d,e^ water	MD average structure in DMSO	M15^d,e^ DMSO
*φ*_1_ [C1′-O-C5-C6]	77.7	86.2	−124.5	−130 ± 9	−129.5	−132 ± 8	−128.8
*φ*_2_ [C1″-O-C3-C4]	97.1	115.7	−154.7	−153 ± 8	−148.0	−152 ± 9	−154.3
*φ*_3_ [O-C1′-O-C5]	−95.1	−107.4	77.6	81 ± 7	81.6	79 ± 8	80.6
*φ*_4_ [O-C1″-O-C3]	93.9	81.0	73.1	81 ± 8	75.9	82 ± 8	81.3
*φ*_1_′ [C4-C5-C6-C7]	50.6	61.2	71.2	73 ± 7	73.2	72 ± 6	66.0
*φ*_2_′ [C2-C3-C4-C5]	118.1	153.2	−162.8	−158 ± 4	−165.0	−161 ± 3	−168.3
*φ*_3_′ [O-C1-C2-C3]	122.6	132.3	−125.7	−129 ± 2	−137.7	−125 ± 3	−97.8
*φ*_4_′ [C12-C13-O-C1]	147.2	100.4	−128.1	−117 ± 2	−100.5	−124 ± 3	−154.7
*φ*_5_′ [C10-C11-C12-C13]	71.2	−173.3	−156.6	−160 ± 3	−170.4	−156 ± 3	−170.1
*φ*_6_′ [C9-N-C10-C11]	−161.5	−63.2	−146.7	−147 ± 1	−141.6	−149 ± 1	−127.8
MAD^f^				4	8	3	11

^a^Values for DFT-optimized initial structure M1 at the *ω*B97X-D/6–31G(d,p) level of calculation, which was used as input in the MD simulation.

^b^Values for structure M12 optimized at the *ω*B97X-D/6–31G(d,p) level using the MD last frame.

^c^X-ray data from [15].

^d^The last structure from the 100 ns MD trajectory was used as the input for geometry optimization.

^e^Optimized structure at *ω*B97X-D-PCM/6–31G(d,p) level (gas phase).

^f^MAD stands for mean absolute deviation.

**Figure 4.**
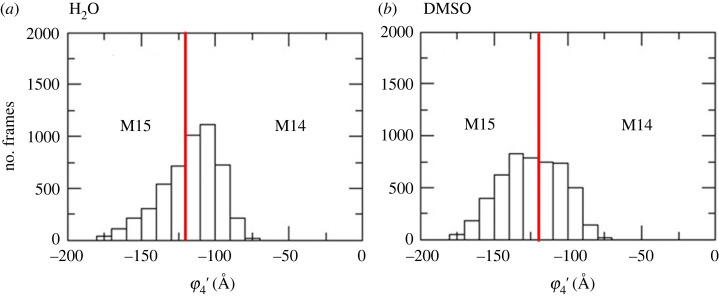
Population distribution as a function of φ_4_′ [C12-C13-O-C1] along 100 ns MD trajectory. 5000 frames were analysed. Conformer M15 is characterized by φ_4_′ < −120° and conformer M14 by φ_4_′ > −120°. (*a*) H_2_O and (*b*) DMSO.

Using the optimized structures for analysis (M12, M14 and M15), we noted that the largest absolute deviation (AD) was for φ_4_′ [C12, C13, O, C1] (approx. 28°). It can be seen from table 1 that the optimized geometries are φ_4_′ approximately −100° and −155° for structures M14 and M15 respectively. These two conformers differ in the intramolecular OH11 … O1 H-bond, which is formed only in M14 (figure 3*b,c*); however, this is not the case along the MD trajectory where the solvent is explicitly included. The average φ_4_′ over the MD trajectories were −117° (water) and −124° (DMSO), which are closer to the X-ray structure (−128°). In the average MD conformation, the OH11 … O1 H-bond is not frequent, with a distance of 3.8 ± 0.7 Å (water) and 4.1 ± 0.8 Å (DMSO). Figure 4 represents the number of conformations (no. frames) as a function of φ_4_′. At first glance, we observed that the conformer with φ_4_′ < −120° (figure 3*c*) dominates, being more evident in DMSO (approx. 90% with average φ_4_′ = −124 ± 3°).

This might be partially interpreted based on the nature of the solvent and solute–solvent interactions, represented by the radial distribution function (g(r)) (electronic supplementary material, figure S2). DMSO acts exclusively as an H-acceptor and forces OH11 to change its conformation and interact with the S = O group, breaking the OH11 … O1 intramolecular H-bond and forcing the X-ray conformation (figure 3*c*). Conversely, H_2_O may act as both an H-donor and an H-acceptor, favouring both conformers (figure 3). The profiles of OH11 … solvent g(r) in DMSO and water suggest a stronger H-bond in DMSO, characterized by a thin and high peak at approximately 2 Å. The OH11 … solvent g(r) in water shows that H_2_O may also act as an H-donor, which explains the conformation with OH11 … O1 H-bond (figure 3*c*, φ_4_′ = −117° ± 2).

Overall, the agreement with the X-ray structure was worse for conformer M15 than M14, despite the absence of the OH-11 … O1 intramolecular H-bond, which does not exist in the solid-state structure. Specifically, φ_4_′ was approximately −155° for conformer M15, which differs from the experimental value by approximately 27°. For conformer M14, φ_4_′ was approximately −100°, which also differed from the observed value by approximately 28°. Nonetheless, significant distortions were predicted for conformer M15 in φ_3_′ and φ_6_′ (table 1). The φ_3_′ and φ_6_′ torsion angles define the conformation around the N(CH_3_) amine and the O-C1-C2-C3 moiety, respectively, and the latter is directly involved in the OH11 … O1 intramolecular H-bond. These results suggest that the OH11 … O1 intramolecular H-bond affects the conformation of the azacyclopentadecyl macrocycle locally.

Another interesting aspect of the AZM conformation is related to the OH at C6. In solution, we observed that the OH6 … N intramolecular H-bond was formed and remained throughout the entire MD trajectory in both solvents. This H-bond was slightly affected by conformational changes, as shown in figure 3. For conformer M15, which is dominant in solution, the OH6 … N distance was 1.646 Å, and for conformer M14 the H-bond distance was 1.780 Å (figure 3). This result shows that the OH11 … O1 H-bond affects the OH6 … N H-bond; that is, if the former is present, the OH6 … N distance is longer (see figure 3*b,c*).

To complete the discussion on the OH … solvent interactions, electronic supplementary material, figure S3 represents g(r) for OH6 and OH12. For OH6, H_2_O acts as an H-donor, and no interaction with DMSO was predicted. This suggests that the OH6 … N H-bond is fairly strong, regardless of the presence of conformations M14 and M15. Conversely, the behaviour of OH12 was similar to that of OH11, with H_2_O acting as the H-donor and H-acceptor and DMSO as the H-acceptor. The use of NMR analysis to monitor conformational changes is addressed in the following paragraphs.

In summary, MD simulations showed that the AZM structure is fairly rigid in solution (regarding heavy atom positions), with some flexibility around the C9-N-C10-C11 and C12-C13-O-C1 moieties induced by OH1 … O1 H-bond formation (see figure 3). Under thermodynamic conditions in the presence of explicit solvent molecules, the OH11 … O1 H-bond is infrequent, mainly in DMSO, which acts only as an H-acceptor. Therefore, three conformers were selected from the MD simulations, named M12, M14 and M15, with the latter two differing by the presence of the OH11 … O1 H-bond (figure 3*b,c*).

All 11 initial structures located on the potential energy curves were further optimized at the *ω*B97X-D/6–31G(d,p) level in the gas phase, followed by harmonic frequency calculations to characterize them as true minimum-energy structures (all frequencies are real).

In addition, we used solid-state X-ray cartesian coordinates [16] to optimize the structure named M13. There are also three new DFT-optimized structures from MD simulations: M12, M14 and M15 (table 1). Therefore, we have 15 distinct optimized molecular structures that faithfully represent the conformational space for the antibiotic AZM. All *ω*B97X-D/6–31G(d,p) optimized structures are shown in electronic supplementary material, figure S4.

**Figure 5.**
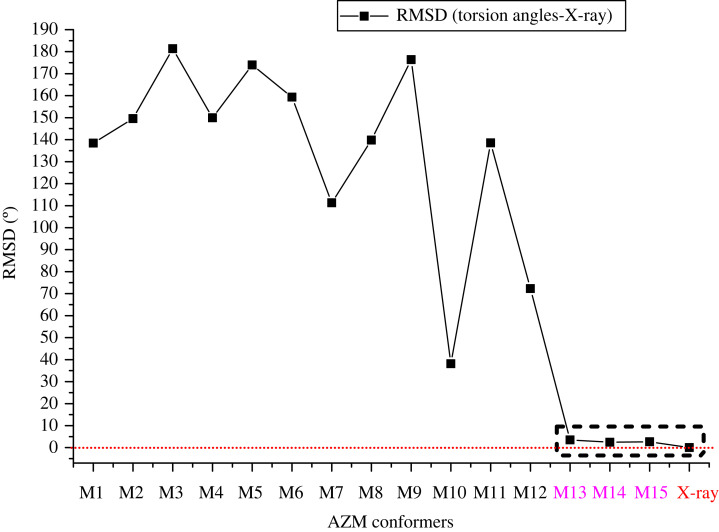
RMSD values concerning X-ray structure [16] for selected *ω*B97X-D/6–31G(d,p) torsion angles used in the relaxed scan curves (figure 2) for all 15 optimized geometries of AZM. The lowest RMSD structures are highlighted showing that the solid-state structure is very close to true minima on the AZM potential energy surface (PES).

The selected optimized torsion angles defined in figure 1 (*φ*_1_, *φ*_2_, *φ*_3_, *φ*_4_, *φ*_1b_, *φ*_2b_), which are considered relevant for structural analysis, are shown in electronic supplementary material, figure S5. There is a large variation in the torsion angles within all distinct optimized structures of AZM, confirming that each of them has a unique minimum energy structure. The RMSD values with respect to the torsion angles of the X-ray structure [16] are shown in figure 5 for all 15 structures. The results show that M13, M14 and M15 resemble X-ray data very well, indicating a great similarity between these DFT-optimized geometries and solid-state structures in terms of the spatial orientation of heavy atoms.

**Figure 6.**
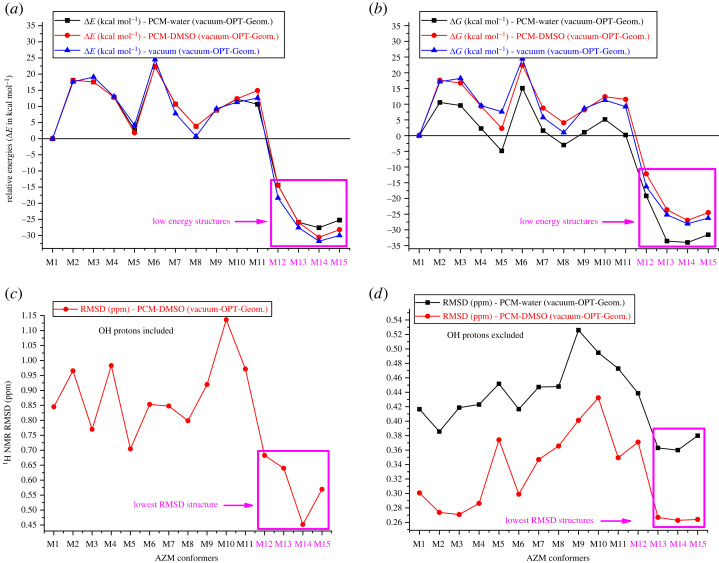
*ω*B97X-D/6–31G(d,p) relative energies in units of kcal mol^−1^ (*a*) ΔE_rel_, (*b*) ΔG_rel_, calculated for all geometries optimized in the vacuum with solvent effects included through PCM single point energy calculations for water and DMSO solvents. These structures are named M1 to M15. B3LYP-PCM/6–31G(d,p) statistical indices (in ppm) values (*c*) RMSD (*d*) RMSD evaluated excluding OH protons. The relative energy values were calculated concerning the M1 structure. Experimental NMR data from [35] were used for the evaluation of statistical indices. No experimental NMR data for OH protons are available in D_2_O.

The relative energy (ΔE_rel_) and Gibbs free energy (ΔG_rel_) results (in kcal mol^−1^) with respect to the M1 structure, evaluated at the *ω*B97X-D-PCM/6–31G(d,p) single-point calculation level (water and DMSO solvents), with geometries optimized in a vacuum, are shown in figure 6*a,b*. For the evaluation of ΔG in solution, gas-phase thermal corrections were employed (this procedure for calculating relative energy values can be justified by the results reported in electronic supplementary material, figure S6, where a comparison of ΔG calculated with geometries optimized in vacuum and PCM-water is presented. The variation in relative energies among all structures is in a wide range of approximately −30 to + 20 kcal mol^−1^, showing a significant change in relative stability among distinct conformers of AZM. The M12, M13, M14 and M15 structures have the lowest energy values, with relative ΔE_rel_ and ΔG_rel_ values in the approximate range of −15 to −30 kcal mol^−1^, and are good candidates as predominant structures in solution from a thermodynamic point of view.

We have now analysed the chemical shift results. The B3LYP-PCM/6–31G(d,p) ^1^H NMR RMSD evaluated using the experimental NMR data from [35] for all optimized structures of AZM are shown in figure 6*c,d* (only CH_n_ type protons were included in the evaluation of RMSD, with the OH protons excluded in figure 6*d*). A large variation was observed as the conformation changed. As in the case of the relative energy values, M12, M13, M14 and M15 structures showed lower RMSD values. Therefore, these structures should be plausible in water and DMSO solutions, according to spectroscopic analysis. The RMSD for the DFT-optimized structure M14 is the lowest among all structures (also the lowest ΔE_rel_ and ΔG_rel_) and therefore is a good candidate to be present in the solution for the two solvents, from this initial analysis.

**Figure 7.**
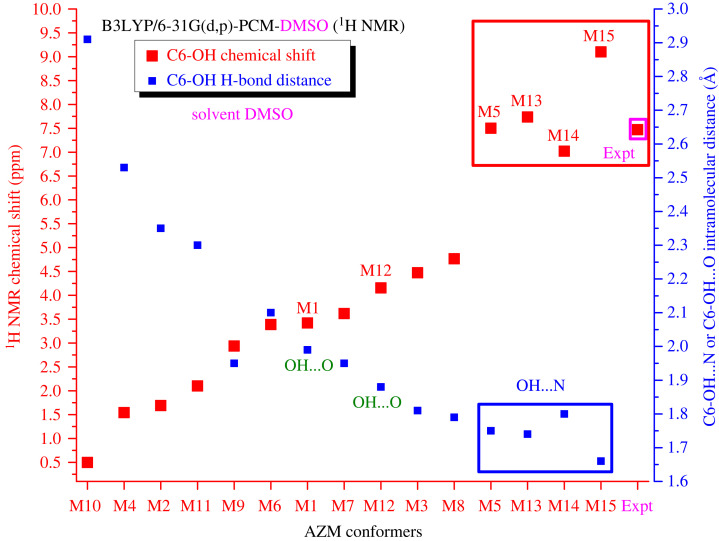
B3LYP-PCM/6–31G(d,p) C6-OH proton ^1^H NMR chemical shifts for all optimized structures (in the vacuum) of AZM (M1 to M15). Experimental data in DMSO-*d6* is also given. C6-OH … N and C6-OH … O H-bond distances are quoted. Experimental ^1^H NMR taken from [35].

One point that should be highlighted is the remarkably high value of the experimental C6-OH proton chemical shift observed in the DMSO-*d_6_* solution (7.47 ppm) [35] compared with the other OH protons. To understand this behaviour, we show in figure 7 the C6-OH proton ^1^H NMR chemical shifts (B3LYP-PCM/6–31G(d,p)) for all optimized structures of AZM, including the experimental data, along with the C6-OH H-bond distance (Å) from the closest N or O atoms (C6-OH … N and C6-OH … O). There was an inverse correlation between the C6-OH chemical shift and the C6-OH … N(O) H-bond distance. This is an interesting result, indicating that the preferred AZM structure in the DMSO-*d_6_* solution should have a short C6-OH … N intramolecular hydrogen bond in solution, preferentially M13, M14 and M15, as shown in figure 7.

Based on the results reported in figure 6 and 7, we will concentrate our attention on the structures M5, M12, M13, M14 and M15. The results for water and DMSO are discussed separately.

**Figure 8.**
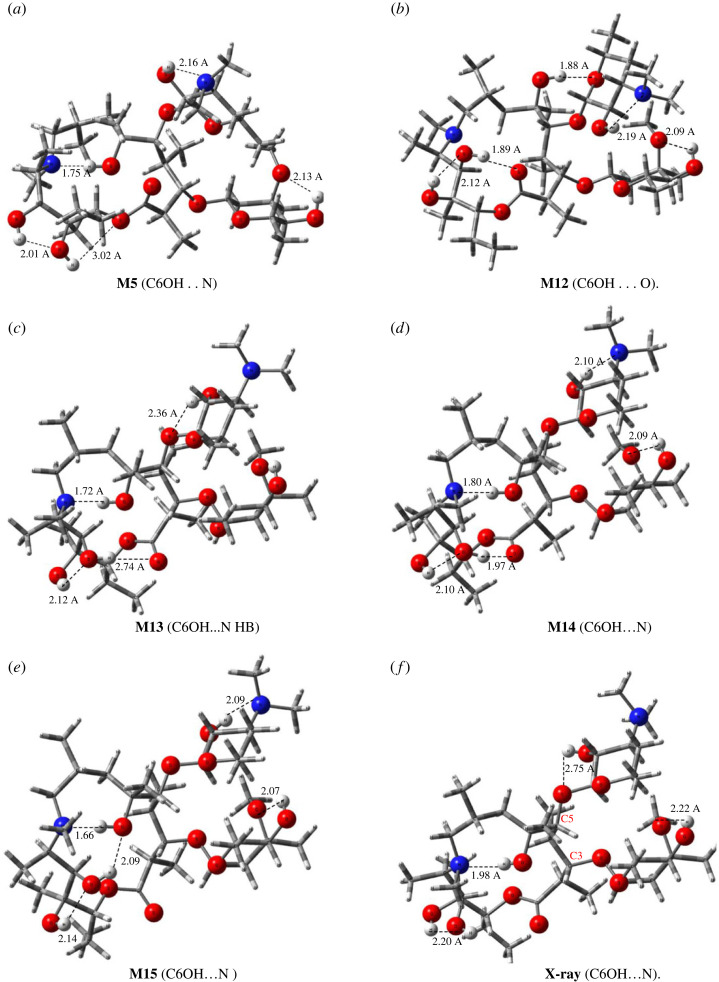
*ω*B97X-D/6–31G(d,p) selected optimized structures located on the PES for AZM molecule. To ease visualization heteroatoms and O-H groups are highlighted. Intramolecular hydrogen bonds (HB) are indicated by a dotted line. X-ray structure (*f*) is also shown. (*a*) **M5** (C6OH…N), (*b*) **M12** (C6OH…O), (*c*) **M13** (C6OH…N HB), (*d*) **M14** (C6OH…N), (*e*) **M15** (C6OH…N) and (*f*) **X-ray** (C6OH…N).

The torsion angles for M1–M15 AZM-optimized structures (electronic supplementary material, figure S5) are distinct, and therefore many equilibrium structures on the potential energy surface correspond to possible different conformations that can be observed in the solution. The strategy we used to identify the predominant structures in solution was an integrated analysis of experimental chemical shift data and DFT-PCM-calculated thermodynamic quantities and NMR chemical shifts, which allowed us to select the conformations shown in figure 8.

It is worth noting that there are no significant differences between torsion angles optimized in vacuum or including solvent effects using the PCM model (electronic supplementary material, figure S6); therefore, single-point DFT-PCM relative energies and NMR calculations (CH_n_ proton chemical shifts) seem adequate at lower computational cost (see electronic supplementary material, figure S7).

Five selected AZM structures (M5, M12, M13, M14 and M15) from the previous analysis (see figure 8), which can be considered the most relevant structures present on the potential energy surface (PES) for AZM, among the 15 structures proposed, were optimized using the PCM model and DMSO and water solvents, followed by the calculation of harmonic frequencies, which enabled us to evaluate the relative Gibbs free energy (ΔG_rel_) values in solution, in addition to ΔE_rel_. These structures were re-optimized by adding five explicit DMSO and water solvent molecules around the target sites for solute–solvent interactions using prior chemical notions to simulate chemically viable solute–solvent interactions at the *ω*B97X-D-PCM/6–31G(d,p) level of calculation, followed by B3LYP-PCM/6–31G(d,p) NMR calculations for the PCM-5DMSO (and 5H_2_O) optimized geometries. This is our best model, which we believe can account for short- and long-range solute–solvent interactions and can produce more accurate thermodynamic and spectroscopic results. A comparative analysis of DFT/6–31G(d,p) relative energies and NMR results calculated with geometries optimized in a vacuum, using the PCM model, and also increasing the basis to a triple-zeta quality 6–311 + G(2d,p) is presented in the (electronic supplementary material, figures S8 (PCM-DMSO) and S9 (PCM-water)), yielding the same results as shown before in electronic supplementary material, figure S7.

**Figure 9.**
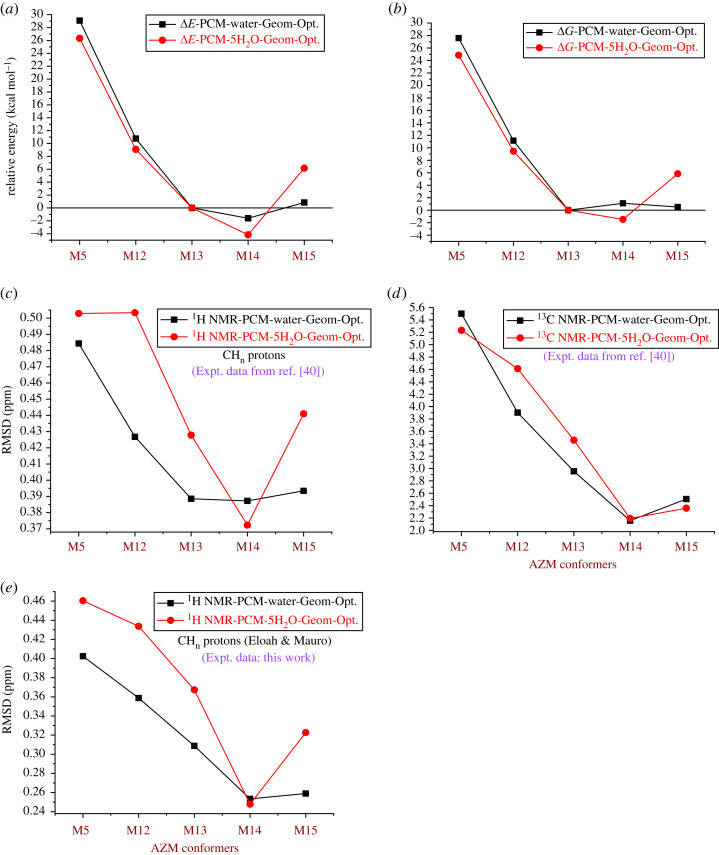
(*a,b*) *ω*B97X-D-PCM/6–31G(d,p) relative energies (ΔE_rel_, ΔG_rel_, in kcal mol^−1^) for selected AZM-optimized structures. B3LYP-PCM/6–31G(d,p) RMSD results are shown in (*c–e*) ^1^H NMR and ^13^C NMR. Calculations using the implicit solvent model (PCM-Water-Geom-Opt.) and five explicit H_2_O molecules (PCM-5H_2_O-Geom-Opt.) are reported.

All theoretical data obtained for geometries optimized using two approaches, the PCM model (PCM-Water) and five explicit H_2_O solvent molecules (PCM-5H_2_O), are shown in figure 9. ^1^H NMR RMSD values were evaluated, including CH_n_ protons only (OH protons were omitted), because no experimental NMR data for OH protons were observed. *ω*B97X-D-PCM/6–31G(d,p) relative energy results, evaluated with respect to the M13 structure, are summarized in figure 9*a,b*, and B3LYP-PCM/6–31G(d,p) ^1^H NMR and ^13^C NMR statistical indices (RMSD in ppm) are shown in figure 9*c–e*. ΔE_rel_ and ΔG_rel_ exhibited similar profiles, and the inclusion of thermal correction did not significantly alter the energy trend. M13 and M14, which are very similar structures differing only in the position of the H2′ H-bond, are the preferred AZM conformations using both the implicit and explicit DMSO solvent models; however, there is a visible preference for M14 when explicit solvent molecules are included in the geometry optimization.

Regarding ^1^H NMR chemical shifts, the PCM-only results indicate that M13, M14 and M15 structures are equally likely to exist in an aqueous solution. However, the inclusion of five explicit H_2_O solvent molecules leaves no doubt of the predominance of the M14 structure, which is also corroborated by ^13^C NMR analysis. Our experimental data (electronic supplementary material, figure S13*e*) were used instead of those reported by Brennan and Barber. [35] We reached the same conclusion. A joint analysis of ΔG_rel_ and RMSD values indicated that M14 was most likely to exist in the water solution.

The theoretical ^1^H NMR chemical shift deviations from the experimental data (in D_2_O) for structures optimized using the PCM model and including five explicit DMSO solvent molecules are reported in electronic supplementary material, figure S10*a* for macrocycle-type protons and in figure S10*b–d* for sugar unit protons. The protons of the sugar units are highlighted in electronic supplementary material, figure S10*a–c* (H1′, H2′, H1″, H2″, H3, H4, H5). The sugar units and macrocyclic protons are shown separately for easy comparison. Although the deviation patterns for sugar units may not reveal a clear preference for a given structure, the macrocycle protons for structures M14 (and M13) showed the smallest deviation. This is consistent with the RMSD results. The analysis in electronic supplementary material, figure S10 reveals which part of the molecule is closest to the observed structure in solution and the protons farthest from the correct position. Obtaining a PCM-5H_2_O optimized structure with ^1^H NMR deviations for all protons sufficiently low (below 0.1 ppm) is a difficult computational task but would lead to an ideally optimized structure in solution. However, we do not believe that further computationally expensive geometry optimizations with tighter convergence criteria would significantly change the conformations that we already have. This would cause only a small enough structural change to tune the ^1^H NMR chemical shifts and lower the deviation from experimental data and statistical index values.

The ^1^H NMR spectrum was recorded using D_2_O solvent; thus, the OH proton signal was not observed; only CH_n_-type chemical shifts have been reported in [35]. Because there are many such protons, the spectrum is too crowded, making it difficult to clearly assign the molecular structure that leads to the best match between the experimental and theoretical spectra. The calculated (PCM-5H_2_O) and experimental ^1^H NMR spectra for the selected protons, as well as the full spectra, are shown in electronic supplementary material, figure S11. The best agreement with the experimental ^1^H NMR profile for the M14 structure following the analysis of RMSD results is not straightforward to see by comparing the experimental and theoretical spectra shown in electronic supplementary material, figure S11. Analysis of the full spectrum (right side of electronic supplementary material, figure S12) was useless.

**Figure 10.**
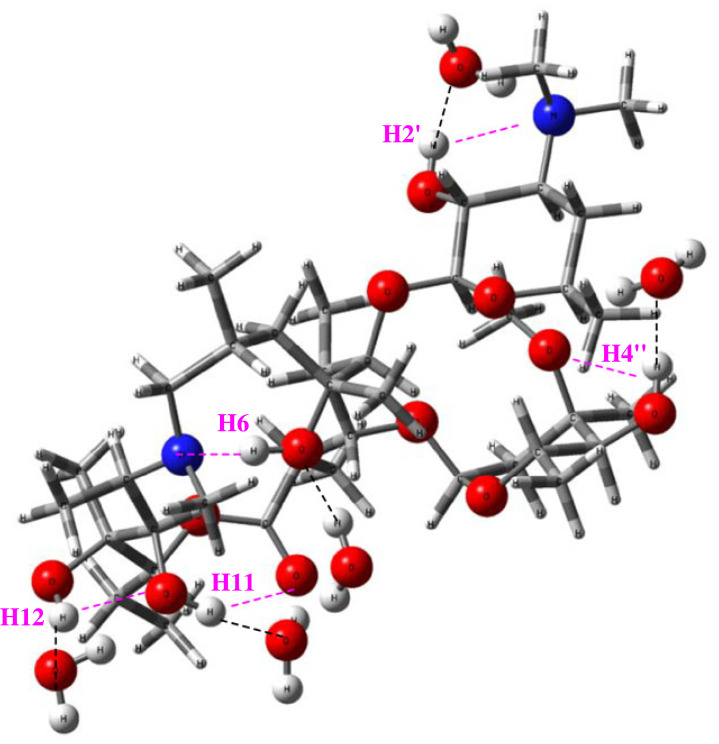
*ω*B97X-D-PCM/6–31G(d,p)-optimized M14-5H_2_O structure of AZM, most probable to exist in aqueous solution. Solute–solvent H-bond distances are indicated by dashed lines. AZM-heavy atoms are highlighted for easy visualization.

The optimized M14 structure, including five explicit H_2_O solvent molecules (named DFT-PCM-5H_2_O), is shown in figure 10, with intermolecular solvent-solute (H_2_O … HO) H-bond and intramolecular solute-solute (OH … N and OH … O) distances and angles given in table 2 (in Å). The N atoms and OH groups are highlighted for ease of visualization. Comparison of implicit (PCM-water) and explicit (PCM-5H_2_O) solvated intramolecular H-bonds for the M14 structure showed that the interaction with water molecules systematically increased the H-bond distances, with the exception of C6-OH … N, which showed a relatively small decrease due to solute–solvent interactions (the increase in the C6-OH … N hydrogen bond angle is also consequence of this fact). The H-bond distances given in table 2 explain the higher thermodynamic stability of M14-5H_2_O with short solvent–solute intramolecular H-bond distances, leading to the formation of stronger H-bond interactions with water solvent molecules. In other words, solvation appears to be more efficient for M14-5H_2_O.

**Table 2.** *ω*B97X-D-PCM/6–31G(d,p) intramolecular O-H … O and O-H … N distances (Å), angles (°) and AZM-H_2_O intermolecular H-bond distances (Å) for AZM structure M14. See figure 1 for the numbering scheme.

intramolecular distance (Å)					
	C2′-OH … N	C4″-OH … O	C6-OH … N	C11-OH … O	C12-OH … O
PCM-only	2.05/123.3	2.11/115.5	1.77/168.4	1.91/151.0	2.08/118.9
PCM-5H_2_O	2.64/100.9	2.51/103.2	1.63/170.6	3.47/94.5	2.29/109.5
intermolecular distance (Å)					
	C2′-OH … O-H_2_	C4″-OH … O-H_2_	C6-OH … H-O-H	C11-OH … O-H_2_	C12-OH … O-H_2_
PCM-5H_2_O	1.79/166.7	1.82/179.5	1.80/170.22	1.70/176.9	1.89/157.2

Finally, according to the PCM-DMSO C6-OH ^1^H NMR chemical shifts results reported in figure 7, the very short C6-OH … N hydrogen bond value of 1.63 Å predicted for structure M14-5H_2_O should yield a corresponding C6-OH chemical shift very much larger (around 10 ppm) that should be easily observed experimentally, as accomplished by Charisiadis *et al*. for phenolic compounds [36]. Intramolecular hydrogen bonds in water are relatively strong, indicating that the solvated AZM structure should not be easily deformed when interacting with the target sites in biological media. The persistence of intramolecular hydrogen bonds in organic compounds in solution is already known and experimentally proven. Exarchou *et al.* showed that the typical hydrogen bond common in the fundamental nucleus of flavonoids (C4O … HO5) remains in solvents such as water, acetone and methanol [37].

The relative energy and NMR data calculated using DMSO (similar to figure 9 for water) are shown in electronic supplementary material, figure S12. It can be seen from the ΔE and ΔG results that the M13, M14 and M15 structures are the preferred AZM conformations using both implicit and explicit DMSO solvent models. On the other hand, structure M14 should be predominant according to ^1^H NMR and ^13^C NMR RMSD values, which is in agreement with the water solution results. As shown in electronic supplementary material, figure S12, the ^1^H NMR RMSD results show a different pattern depending on the inclusion or absence of OH protons in the analysis. Our results show that ^13^C NMR chemical shifts are less sensitive to solvent effects than ^1^H NMR (influenced by the spatial orientation of OH protons around the solute). Therefore, a comparative analysis of ^13^C NMR chemical shifts may be useful for the conformational analysis of heavy atoms.

**Figure 11.**
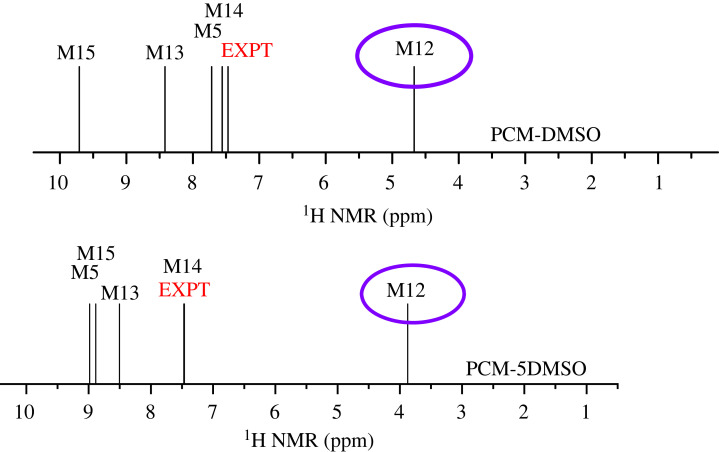
B3LYP-PCM/6–31G(d,p) ^1^H NMR chemical shift for C6-OH proton for relevant optimized structures of AZM. The circled structures exhibit a too-small value of C6-OH chemical shift compared with the experimental data.

A remarkable feature of the experimental ^1^H NMR spectrum (in DMSO-*d_6_*) is the observation of a noticeably large chemical shift value of 7.47 ppm (the largest experimentally value) assigned to a C6-OH proton. This value can be used as a reference for comparison with our theoretical ^1^H NMR results to select the most probable AZM structure to be present in the DMSO solution. Figure 11 shows the experimental and theoretical C6-OH chemical shift values calculated using the two previously defined approaches, PCM-DMSO and PCM-5DMSO, for the relevant AZM structures. The structure M12, which shows a very large deviation from the experimental data, is highlighted in the purple circle (underestimated by more than 3.5 ppm) and can be ruled out to exist in the DMSO solution. The corresponding chemical shift values (less than 4 ppm) were mixed with ^1^H NMR values for the CH_n_ protons and did not contribute to the observed C6-OH proton NMR profile. Our best model (PCM-5DMSO) predicted structure M14 as the preferred one in the DMSO solution so far as C6-OH proton NMR analysis is concerned. This is in good agreement with the results found in the aqueous solution, indicating that the C6-OH proton NMR signal can be used to identify the preferred AZM structure in the solution.

At this point, it is important to highlight the role of intramolecular interactions in the thermodynamic discussion. Figure 9*b* shows the data in PCM and in the solvation model PCM + 5H_2_O. The data reveal that the thermodynamic preference of the M14 structure only becomes evident when explicit solvent molecules are added to the model. This is due to the fact that the same intramolecular interaction (C6-OH … N) which allows a good agreement with the experimental signal of C6-OH also causes a decrease in conformational entropy, leading to $\Delta G_{\mathrm{rel}}^{\mathrm{PCM}}>0$ for M14 in figure 9*b*. When considering explicit solvation, entropic contributions of the solvent overcome the drop in entropy caused by the intramolecular interaction C6-OH … N, leading to $\Delta G_{\mathrm{rel}}^{\mathrm{PCM}+5H_{2}O}<0$ for M14 in figure 9*b*.

Electronic supplementary material, figure S13 shows the B3LYP-PCM/6–31G(d,p) ^1^H NMR spectra for the optimized structures shown in figure 8, along with the experimental spectrum (DMSO-*d_6_*). Only chemical shifts of OH protons are given. The full spectrum, including the CH_n_ protons, is too crowded and difficult to analyse. Two sets of NMR calculations are presented using only the PCM model (PCM-DMSO) and with five DMSO molecules (PCM-5DMSO) for geometry optimization. The visible dependence of the ^1^H NMR profile on the specific molecular structure was observed, and a comparison with the experimental pattern appears to be a natural criterion. The effect of including explicit DMSO solvent molecules on the ^1^H NMR spectrum of OH protons is quite remarkable, except for the C6-OH proton, which forms a strong H-bond with the macrocycle N atom and is not very sensitive to the inclusion of explicit solvent molecules.

Combining ΔG_rel_, RMSD and ^1^H NMR chemical shifts for OH protons result in an unambiguous conclusion that structure M14 is most likely to exist in DMSO solution, similar to aqueous media. Modelling solvent effects could be improved by increasing the number of solvent molecules in DFT geometry optimization, but this would be computationally inviable. Nevertheless, our model was reliable and sufficient to predict reliable relative energies and chemical shift values for use in the conformational analysis of organic molecules.

Finally, the best-solvated structure, M14-5DMSO is shown in electronic supplementary material, figure S14, where the solute–solvent distances (and C6-OH … N H-Bond) are indicated. AZM heavy atoms are highlighted for easy visualization. The intramolecular (O-H … O and O-H … N) and solute-solvent (O-H … O=S) distances (Å) for the PCM-DMSO and PCM-5DMSO optimized M14 structure are given in electronic supplementary material, table S2. The inclusion of explicit solvent molecules increases the intramolecular O-H … O and O-H … N distances owing to strong interactions with DMSO (see electronic supplementary material, table S1). However, the C6-OH … N H-bond is unchanged because steric hindrance hinders interactions with solvent molecules, similar to the water solution behaviour.

## 4. Conclusion

In this work, we used a quantum chemical method (DFT) to carry out a conformational analysis of AZM, with solvent effects described by a continuum model (PCM), including five explicit solvent molecules (water and DMSO). Through the calculation of the relaxed potential energy scans, 11 minimum energy structures were found. Using MD simulation frames and solid-state structures as inputs for DFT geometry optimization, four other minima were obtained, totaling 15 true equilibrium structures located on the PES for AZM (named M1–M15).

To select the most probable structures among the 15 plausible conformers of AZM found in a macroscopic experimental solution, DFT-PCM relative energies and NMR chemical shifts were used as the thermodynamic and spectroscopic criteria. Structure M14 showed the best match between the experimental (DMSO) and theoretical (PCM including five explicit solvent molecules) ^1^H NMR profiles, being almost degenerate with M13 structure on a thermodynamic basis; therefore, these conformers are predicted to be good candidates for observation in DMSO solution. In water solution, both relative energies and RMSD values for ^1^H NMR (CH_n_ protons only) and ^13^C NMR chemical shifts also predicted structure M14 as preferred in water solution, which is very similar to the solid-state structure regarding heavy atom configuration. This is very likely due to the specific molecular structures that interact with target sites in biological media. Therefore, to conduct efficient molecular modelling studies on drug–receptor interactions, it is important to choose a drug input structure that represents the predominant conformation present in an aqueous solution, not necessarily the solid-state structure, which is a common procedure.

Finally, we showed in this work that large molecules with a large number of possible conformations may assume a predominant spatial orientation in solution, as strongly indicated by our DFT thermodynamic and spectroscopic NMR results, which should be used in further drug-interaction studies.

## Data Availability

The datasets supporting this article have been uploaded to Dryad: https://doi.org/10.5061/dryad.7d7wm380p [38]. The data are provided in electronic supplementary material, [39].
